# Left ventricular twist mechanics and its relation with aortic stiffness in chronic kidney disease patients without overt cardiovascular disease

**DOI:** 10.1186/s12947-016-0053-8

**Published:** 2016-03-09

**Authors:** Samir Sulemane, Vasileios F. Panoulas, Klio Konstantinou, Athanasios Bratsas, Frederick W. Tam, Edwina A. Brown, Petros Nihoyannopoulos

**Affiliations:** 1 Imperial College London, National Heart and Lung Institute, Sydney Street, SW6 3NP London, UK; 2 Imperial College Healthcare NHS, Hammersmith Hospital, Ducane road, W12 0HP London, UK; 3 Imperial College Renal and Transplant Centre, Hammersmith Hospital, Ducane road, W12 0HP London, UK

**Keywords:** Chronic kidney disease, LV twist, Speckle tracking echocardiography, Arterial stiffness, Aortic pulse wave velocity

## Abstract

**Background:**

Recent studies hypothesized left ventricular (LV) twist as a potential biomarker for evaluation of sub clinical myocardial disease, however its relationship with aortic stiffness has yet to be investigated. Chronic kidney disease (CKD) has been identified as a risk factor for both myocardial and arterial disease. As such we sought to explore the relationship between aortic stiffness and LV twist in CKD patients without known cardiovascular disease (CVD).

**Methods:**

In this prospective, observational study we enrolled 106 CKD patients (Stages 1 to 5) with normal LVEF as assessed by conventional echocardiography. Aortic stiffness was measured using aortic pulse wave velocity (aPWV). We defined increased aPWV as ≥10 m/s. LV Twist was measured using two-dimensional speckle tracking echocardiography.

**Results:**

Patients with increased aPWV had higher LV twist (*p* = 0.002) but similar LVEF (*p* = 0.486). Aortic PWV correlated crudely with age (*p* < 0.001), the presence of diabetes (*p* < 0.001), hypertension (*p* < 0.001), eGFR (*p* < 0.001), LVMI (*p* = 0.01), e/e’ (*p* < 0.001) and LV twist (*p* = 0.003). In multivariable analyses after adjusting for age, gender, cardiovascular risk factors and hypertensive medication, aPWV was independently associated with LV twist (β = 0.163, *p* = 0.025).

**Conclusions:**

Aortic stiffness independently associates with LV Twist in asymptomatic CKD patients. These findings suggest a close interaction between LV twist mechanics and arterial remodeling even before CVD becomes clinically relevant.

## Background

Recent data suggests that chronic kidney disease (CKD) patients develop both arterial and myocardial dysfunction at an early stage of the disease. Wang and colleagues identified that increased arterial stiffness is evident as early as CKD stage 2 [1]. Even though obstructive epicardial atherosclerotic disease is not an uncommon finding in patients with advanced CKD, early atherosclerotic changes in the macro- and microvasculature result in arterial stiffness that subsequently leads to structural myocardial disease [2, 3]. These pathophysiological features are manifested by a high risk of lethal arrhythmias, congestive heart failure and stroke [3]. Extensive research on methods for assessing arterial stiffness has led to a consensus that aortic pulse wave velocity (aPWV) should be regarded as the ‘gold standard’ [4]. Aortic PWV has been validated in a variety of clinical settings including in CKD [5–7].

Panoulas et al identified Left Ventricular (LV) twist as a potential marker of sub clinical LV systolic dysfunction in CKD patients with normal ejection fraction, as measured by conventional 2D echocardiography [8]. Abnormal LV twist values were seen as early as CKD stage 3. LV twist refers to the systolic twisting motion resulting from basal clockwise rotation and apical counter-clockwise rotation (when viewed from the apex) [9]. Previous data attribute up to 40 % of LV stroke volume to ventricular twist dynamics [10]. Furthermore LV twist has proven to be a more sensitive marker of subtle myocardial dysfunction when compared with conventional echocardiographic methods, namely LV ejection fraction (LVEF) [11]. Twist mechanics can be accurately assessed using speckle-tracking echocardiography (STE), which has been validated against magnetic resonance imaging (MRI) and sonomicrometry [12].

To date, no study has explored the relationship between arterial stiffness and LV twist mechanics. As CKD has been identified as a risk factor for both arterial and myocardial disease we aimed to explore this association in this high-risk patient cohort.

## Methods

### Study population

A total of 123 consecutive patients with CKD stages 1 to 5 were enrolled from Imperial College Healthcare NHS Trust renal outpatient clinics between 2011 and 2014. Chronic kidney disease was defined on the basis of impaired eGFR plus microalbuminuria, present on at least two occasions over three months or more. Patients with: clinical or echocardiographic evidence of LV systolic dysfunction, significant valvular abnormalities (moderate or severe), presence of atrial fibrillation or flutter, pulmonary hypertension, congenital heart disease, cardiomyopathy, pericardial disease or inadequate echocardiographic acoustic windows were excluded from this study. Written informed consent was obtained from all participants. The study was approved by the UK National Research Ethics Committee Service (REC 10/H0704/81).

### Data collection

The collection of anthropometric data included height (cm), weight (kg), body mass index (BMI, kg/m2) and body surface area (BSA, g/m2). Using a structured questionnaire and medical notes review we collected the following data: systolic and diastolic blood pressure, both measured in the sitting position in mmHg, hypertension (defined as SBP ≥ 140 mmHg and/or DBP ≥ 90 mmHg or on antihypertensive treatment), diagnosis of diabetes, treated hypercholesterolaemia (use of statin, fibrate or ezetimibe), family history of ischaemic heart disease, smoking status (current, ex, never) and detailed list of current medication. Biochemical results were obtained from the most recent renal clinic review (within 1 month of recruitment) provided that there was no evidence of superimposed acute kidney injury during the time of blood sampling. The value of eGFR was calculated using the four-variable equation in the Modification of Diet in Renal Disease study [13].

### Echocardiographic assessment

Transthoracic echocardiography was performed using a commercially available system (Vivid 7, GE Vingmed Ultrasound, Horten, Norway) by a single, accredited echocardiographer according to a standardised protocol. All echocardiographic parameters were measured offline in batches by one observer blinded to clinical and outcome data. Interventricular septum thickness, posterior wall thickness, LV mass index (LVMI), tissue Doppler imaging S’ wave (TDI S’), left atrial (LA) dimension and LVEF were measured according to the recommendations of the American Society of Echocardiography [14].

### 2-dimensional speckle tracking echocardiography

Speckle tracking analysis was performed offline by the customised software for Vivid (2D-strain EchoPac PC v.7.0.1, GE Healthcare, Horten, Norway). Basal and apical parasternal short axis views were recorded for each patient. The basal plane was defined as that allowing visualization of the mitral valve with the cross-section as circular as possible. The apical plane was acquired distally to the papillary muscles [15]. LV twist was calculated as the net difference in peak systolic rotational strain between the six basal and six apical segments.

While acquiring two-dimensional images we kept the focus position at intermediate depth and adjusted the sector depth and width to include little but the region of interest. Furthermore, the sampling region of interest was adjusted to ensure that most of the wall thickness was incorporated in the analysis avoiding the pericardium. Frame rate was between 60 frames/s and 80 frames/s.

### Arterial stiffness parameters

On the same day, around 10 min after performing the echocardiogram, blood pressure was measured with the subjects in supine position. Pressure waveforms were recorded on the radial, carotid and femoral arteries using applanation tonometry [16]. Carotid-femoral aPWV was calculated using a commercially available device (SphygmoCor, Pulse Wave Analysis System, AtCor Medical), with a high-fidelity Millar strain-gauge transducer (Millar Instruments, Houston, TX) as described previously [6]. Two separate operators conducted the measurements with coefficient of variation of <10 %.

Previous studies have demonstrated that a aPWV cut off of 10 m/s has been shown to provide risk stratification and prognostic value in CKD patients [17]. Therefore, in our study we defined increased aPWV as ≥10 m/s.

### Reproducibility

In order to test inter and intra-observer variability for both LV Twist and aPWV indices, we reassessed the measurements on a sub-group of 20 randomly selected patients. Shortly after the first assessment a second operator, who was blinded to the results, analysed aPWV and LV twist. Subsequently the first operator re-measured the same indices.

#### Statistical analysis

Statistical analysis was performed using SPSS software, version 22 (IBM, Armonk, NY). Variables were tested for normality by the Kolmogorov-Smirnov test. Firstly, analysis was performed by dividing the patients into 2 groups; one with aPWV < 10 m/s and the other with PWV ≥ 10 m/s. Differences between the 2 groups were analyzed using chi-square test for categorical data, *t*-test for continuous normally distributed data and Wilcoxon rank sum test for continuous non-normally distributed data. Correlations were assessed for normal and non-normal variables using Pearson’s and Spearman’s coefficients respectively. Univariate and multivariate analyses were used to evaluate the relationship between, arterial stiffness and a range of variables including general demographics, cardiovascular risk factors, conventional echocardiographic and speckle tracking parameters including LV Twist. Intraclass correlation coefficients (ICC) and Bland-Altman plots were determined to assess the inter-operator and intra-operator variability of speckle tracking and arterial stiffness measurements. The criterion for statistical significance used was a *P*-value of ≤0.05 to a 95 % confidence interval.

## Results

### Inter- and intra-operator variability of speckle tracking and arterial stiffness parameters

Inter-operator variability (*n* = 20): the ICC for LV twist 0.80, *p* < 0.001.

Intra-operator variability (*n* = 20): the ICC for LV twist 0.89, *p* < 0.001.

**Fig. 1:**
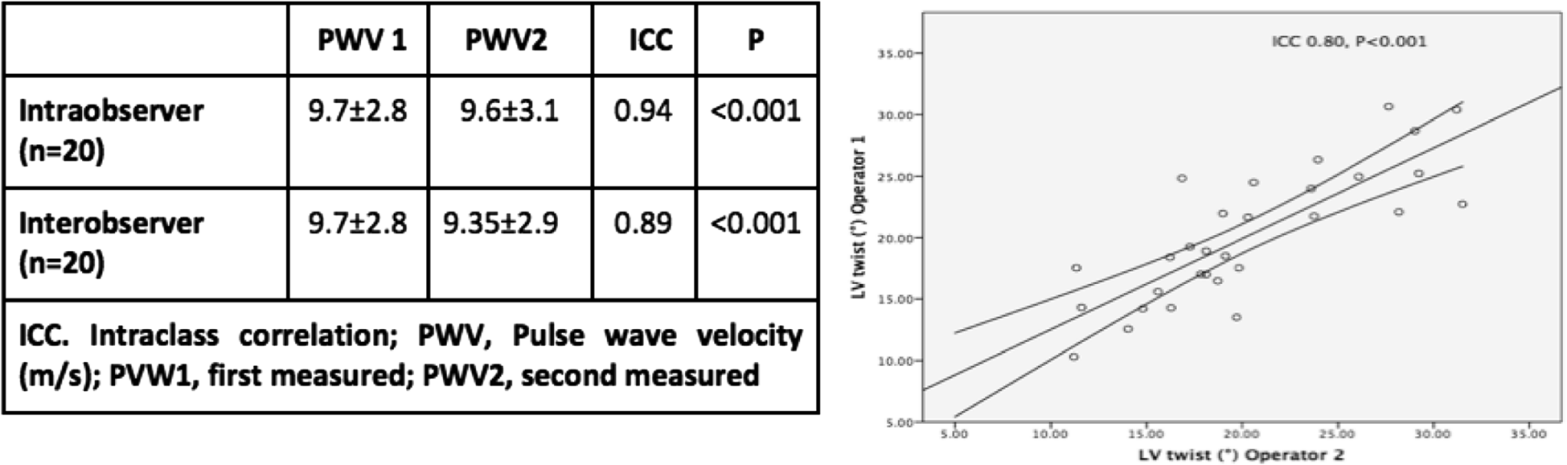
Intra-observer and inter-observer strength of agreement of aPWV (*left*) and Inter-class correlation of LV twist (*right*)

The strength of agreement for intra-observer and inter-observer measurements of aPWV is shown in Fig. 1.

**Fig. 2:**
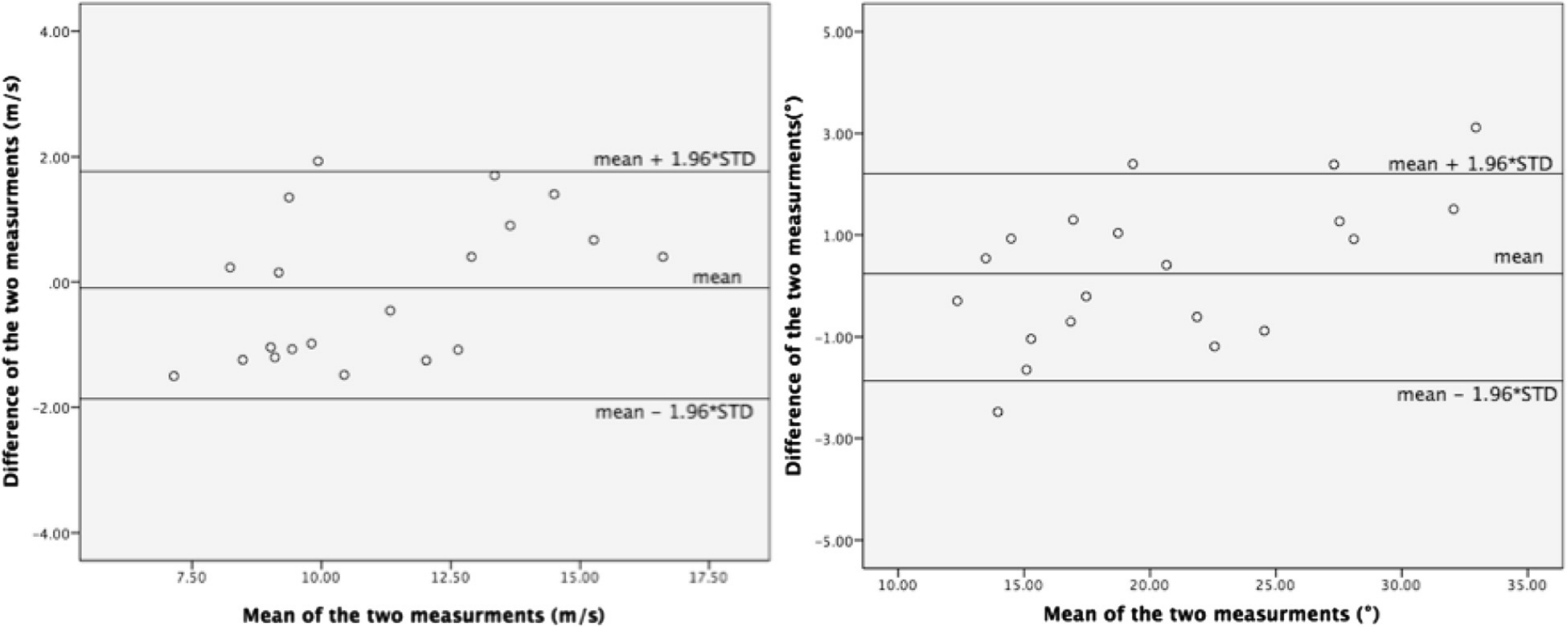
Bland-Altman plot showing inter-observer variability of aPWV (*left*) and LV Twist (*right*)

Figure 2 is a Bland-Altman plot showing inter-observer variability of aPWV and LV Twist.

### Feasibility of obtaining 2D speckle tracking deformation indexes

**Fig. 3:**
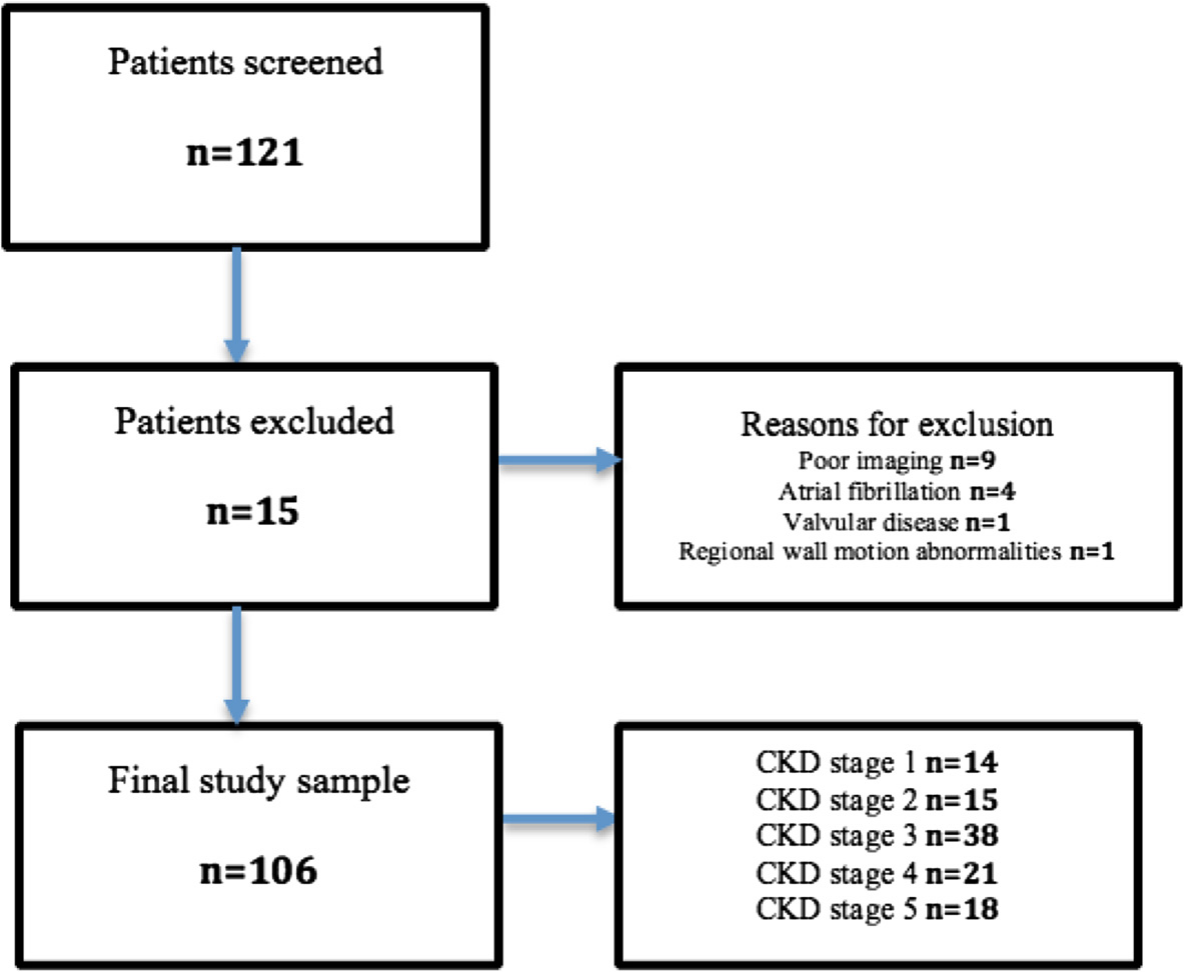
Feasibility in obtaining speckle tracking deformation parameters

Deformation parameters could not be quantified in 15 patients due to sub-optimal echodiagnostic windows (*n* = 9), atrial fibrillation (*n* = 4) valvular abnormalities (*n* = 1) and regional wall motion abnormalities (*n* = 1). A total of 106 patients were finally included in the study (Fig. 3).

### Clinical and echocardiographic characteristics

95 CKD patients (89.6 %) underwent renal biopsy to determine the cause of their CKD. The most common CKD aetiology was diabetes (*N* = 30), followed by vasculitis (*N* = 18), post-renal causes (*N* = 10), IgA nephropathy (*N* = 7), focal glomerulosclerosis (*N* = 6), membranous glomerulonephritis (*N* = 5), hypertension (*N* = 4), adult polycystic kidney disease (*N* = 3), renovascular disease (*N* = 3), and minimal change disease (*N* = 3). In six patients, aetiology was unknown.

**Fig. 4:**
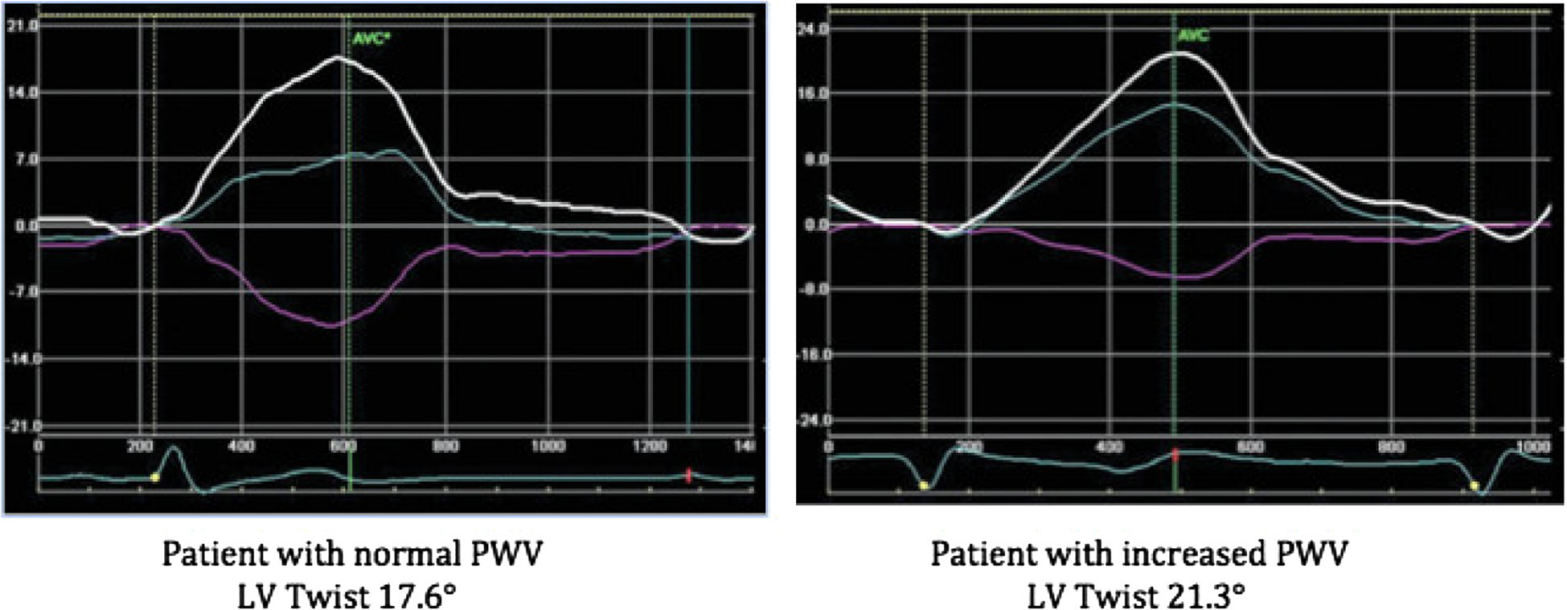
LV twist in a patient with normal and increased PWV

Patients were stratified in two different groups according to aPWV: normal aPWV <10 m/s, increased aPWV ≥ 10 m/s. Table 1 shows patients clinical characteristics. Participants with increased aPWV were more likely to have diabetes (*p* < 0.001) and hypertension (*p* < 0.001) and lower eGFR (*p* < 0.001). Table 2 displays echocardiographic parameters across the two groups. Patients with increased aPWV had lower TDI S’ (*p* = 0.03), global longitudinal strain (GLS) (*p* = 0.002), higher LVMI (*p* = 0.01), E/e’ (*p* = 0.01), LV Twist (*p* < 0.001)- Fig. 4 but similar LVEF (*p* = 0.482). Table 3 shows the variation in LV Twist and in aPWV as we move from early to late CKD stages.

**Table 1:** Clinical characteristics according to aPWV groups

	Normal PWV <10 m/s	Increased PWV ≥ 10 m/s	*p*-value
	*n* = 64	*n* = 42	
Clinical demographics			
Age	51.2 ± 14	63 ± 12	<0.001*
Male gender (%)	45.4	41.2	0.455
BSA (g/m2)	1.88 ± 0.21	1.87 ± 0.22	0.906
Cardiovascular risk factors			
BMI (g/m2)	26.9 ± 5	27.8 ± 5.1	0.367
eGFR (mL/min/1.73 m^2^)	48.95 ± 20.1	30.5 ± 15.4	<0.001*
Systolic BP (mmHg)	127.2 ± 20.4	140.5 ± 18.5	0.01*
Diastolic BP (mmHg)	77.9 ± 10.6	79.5 ± 10.5	0.451
Diabetes (%)	20.3	71	<0.001*
Hypertension (%)	62.5	82.8	<0.001*
Family history of IHD (%)	29.7	26.8	0.755
Smoking status (%) Current			
Current	6.3	9.8	0.295
Ex	9.4	9.8	
Never	45.3	34.1	
Medication			
Aspirin (%)	28	37	0.367
Clopidogrel (%)	2	7	0.135
ACE-I (%)	34	69	<0.001*
ARBs (%)	53	51	0.858
Beta-blockers (%)	16	22	0.416
CCB dihydropyridine (%)	25	38	0.151
Loop diuretic (%)	22	34	0.112
Thiazide diuretic (%)	2	10	0.06
Any antihypertensive (%)	40.1	87.2	
Statins (%)	61	80	0.110
Prednisolone (%)	22	10	0.125
Metformin (%)	9	18	0.081
Gliclazide (%)	9	12	0.649
Insulin (%)	8	41	<0.001*

*BSA* body surface area; *BMI* body mass index; *GFR* glomerular filtration rate, *CKD* chronic kidney disease; *BP* blood pressure; *ARB* angiotensin II receptor blocker; *CCB* calcium channel blocker; *IHD* ischemic heart disease; *ACE-I* angiotensin-converting enzyme inhibitor; *ARB* angiotensin II receptor blocker

*Statistically significant difference between the two groups

**Table 2:** Echocardiographic characteristics according to aPWV groups

	Normal PWV <10 m/s	Increased PWV ≥ 10 m/s	*p*-value
	*n* = 64	*n* = 42	
Conventional echocardiography			
Simpsons biplane EF (%)	62.9 ± 5	61.3 ± 4.7	0.48
TDI S’ – septal (cm/s)	10.6 ± 2.4	8.6 ± 1.9	0.03*
LVMI (g/m^2^)	63.7 ± 19	78.4 ± 22	0.01*
RWT	0.44 ± 0.09	0.51 ± 0.12	0.001*
E/e^’^ (average)	8.9 ± 3.7	10.8 ± 4.2	0.01*
LA (cm)	3.68 ± 0.58	3.9 ± 0.63	0.001*
Diastolic dysfunction (%)			
Not present	40.6	22.5	0.001*
Type 1	50	40	
Type 2	9.4	34.9	
LV Twist (°)	19.8 ± 5.4	23.5 ± 5.7	<0.001*
GLS(%)	−19.6 ± 3.3	−17.1 ± 2.9	0.002*
GCS(%)	−23.3 ± 4.3	−22.8 ± 4.9	0.159

*LVMI* left ventricular mass index; *RWT* regional wall thickness; *EF* ejection fraction; *LV* left ventricle, *GLS* Global longitudinal strain; *GCS* Global circumferential strain

*Statistically significant difference between groups

**Table 3:** Echocardiographic and arterial stiffness parameters across CKD stages

	CKD stage 1/2	CKD stage 3	CKD stage 4/5	*p* value^a^	Adjusted *p* value^b^
Simpsons biplane EF (%)	63.7 ± 5.5	62.3 ± 4.9	62.5 ± 4.9	0.617	0.838
LVMI (g/m^2^)	60.5 ± 20	70 ± 19.8	75.6 ± 24.1	0.003^▪^	0.085^▪^
Concentric remodelling (%)RWT > 0.42	31	52.6	72.3	<0.001^‡▪•^	<0.001^‡▪•^
E/e’ (average)	6.96 ± 2.25	9.41 ± 3.87	9.92 ± 3.96	<0.001^‡ ▪^	0.013^‡ ▪^
TDI S’ - septal (cm/s)	11 ± 2.7	10.6 ± 3.7	9.1 ± 2.3	0.02^▪•^	0.03^▪•^
LV Twist (°)	18.5 ± 4.4	19.9 ± 5.4	24.6 ± 5.4	<0.001^‡▪•^	<0.001^‡▪•^
PWV(m/s)	7.7 ± 2	10.3 ± 3.3	12.5 ± 4.2	<0.001^‡▪•^	0.001^‡▪•^

*LVEF* left ventricular ejection fraction; *LVMI* left ventricular mass index; *RWT* regional wall thickness; *LV* left ventricular; *PWV* pulse wave velocity

^a^Statistically significant difference between: ^‡^CKD stage 1/2 and CKD stage 3, ^▪^CKD stage 1/2 and CKD stage 4/5, ^•^CKD stage 3 and CKD stage 4/5

^b^Adjusted for age, gender, diabetes, SBP, BMI, treated hyperlipidaemia, eGFR and family history of ischaemic heart disease

### Univariate association of aPWV with demographics, risk factors and echocardiographic parameters

**Fig. 5:**
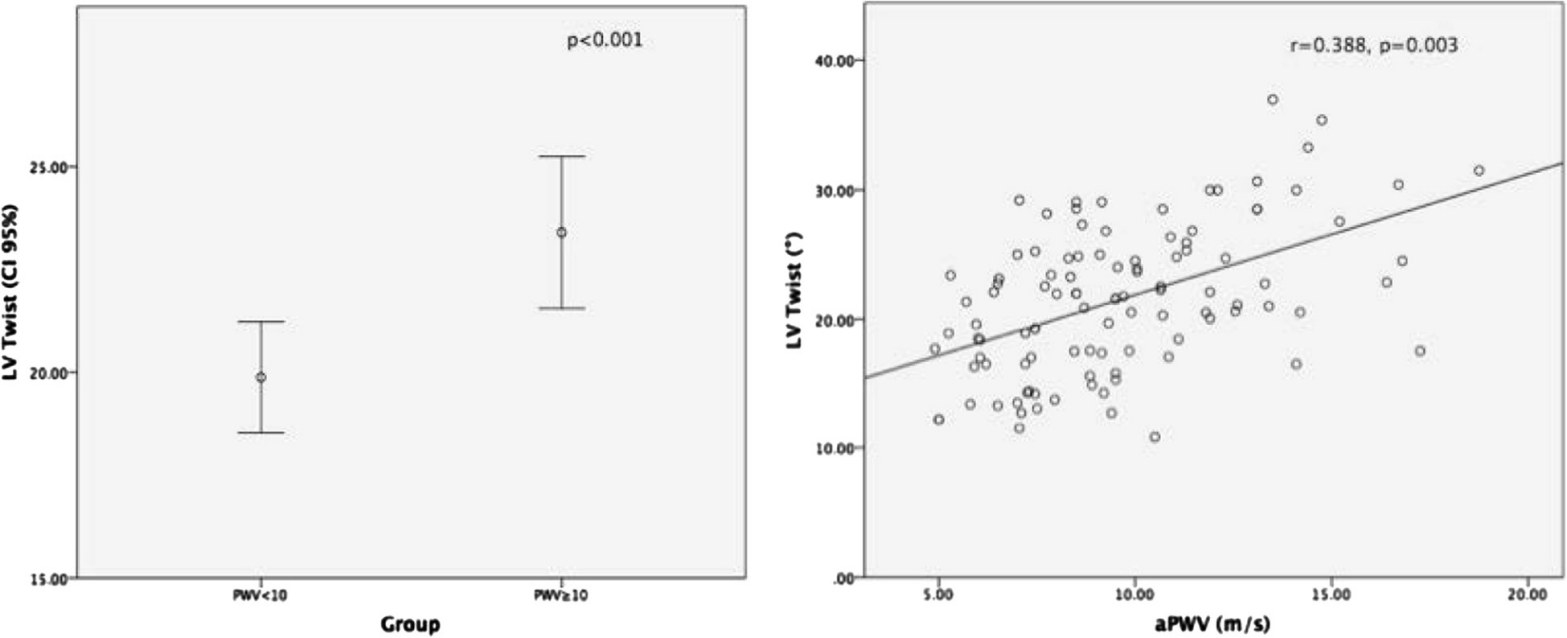
Increased LV twist in patients with preserved and increased aPWV (*left*); Scatter plot correlating aPWV (m/s) and LV Twist (°) (*right*)

Table 4 displays univariate correlations of aPWV with clinical and echocardiographic parametres. Aortic PWV correlated crudely with age (*p* < 0.001), the presence of diabetes (*p* < 0.001), hyperlipidemia (*p* = 0.013), peripheral SBP (*p* < 0.001), hypertension (*p* < 0.001), eGFR (*p* < 0.001), LVMI (*p* < 0.001), E/e’ (*p* < 0.001), TDI S’ (*p* = 0.03), GLS (*p* = 0.01) and LV twist (*p* = 0.003). Figure 5 is a scatter plot demonstrating the correlation between aPWV and LV Twist.

**Table 4:** Univariate and multivariate analysis of aPWV

	Crude		Adjusted^a^		Adjusted^b^	
	Standardized β	*P* -value	Standardized β	*p*-value	Standardized β	*p*-value
Age	0.635	<0.001*	0.492	<0.001*	0.467	<0.001*
Gender	−0.172	0.079	−0.019	0.654	−0.013	0.691
BSA (g/m2)	0.27	0.187	0.031	0.238	0.345	0.459
Hemoglobin (g/L)	−0.150	0.131	−0.059	0.481	−0.098	0.221
Hyperlipidemia	0.242	0.013*	0.118	0.144	0.065	0.433
Peripheral systolic BP (mmHg)	0.397	<0.001*	0.185	0.024*	0.169	0.059
Diabetes (%)	0.589	<0.001*	0.555	<0.001*	0.532	<0.001*
Hypertension (%)	0.308	<0.001*	0.294	0.013*	0.158	0.050*
BMI (g/m2)	0.055	0.579	−0.156	0.063	−0.133	0.118
eGFR (mL/min/1.73 m^2^)	−0.410	<0.001*	−0.259	0.003*	−0.234	0.008*
Echocardiographic parameters						
Simpsons biplane EF(%)	−0.117	0.237	−0.036	0.646	−0.094	0.634
TDI S’ - septal (cm/s)	0.186	0.03 *	0.120	0.058	0.101	0.093
E/e^’^(average)	0.387	<0.001*	0.192	0.022*	0.113	0.089
LVMI (g/m^2^)	0.379	<0.001*	0.165	0.03*	0.143	0.042*
LV Twist(°)	0.388	0.003*	0.181	0.019*	0.163	0.025*
GLS(%)	0.300	0.01*	0.186	0.09	0.060	0.130
GCS(%)	0.046	0.197	0.018	0.458	0.010	0.634

Standardized coefficients (β) refer to how many SDs a dependent variable (aPWV) will change, per standard deviation increase in the predictor variable. *n* = 106

*statistically significant

*CKD* chronic kidney disease; *BSA* body surface area; *BP* blood pressure; *LVMI* left ventricular mass index; *eGFR* estimated glomerular filtration rate; *EF* ejection fraction; *LV* left ventricular

^a^Adjusted for age, gender, diabetes, SBP, BMI, treated hyperlipidaemia, eGFR and family history of ischaemic heart disease

^b^Adjusted for factors in Model 2 plus antihypertensive medication (b-blockers, dihydropyridine calcium channel blockers, ACE inhibitors, angiotensin II receptor blockers) and prednisolone

### Multivariable analysis

In the linear regression model, aPWV was independently associated with age (β = 0.467, *p* < 0.001), diabetes (β = 0.532, *p* < 0.001), hypertension (β = 0.158, *p* = 0.05), eGFR (β = -0.234, *p* = 0.008), LMVI (β = 0.143, *p* < 0.042) and LV twist (β = 0.163, *p* = 0.025) Table 4.

## Discussion

To our knowledge this is the first study to associate aPWV, a marker of aortic stiffness, with LV twist, a myocardial deformation index. The independent association between LV twist and aPWV in asymptomatic CKD patients, suggests an interplay between arterial and ventricular mechanics early on in the cardiovascular disease continuum.

Arterial remodeling has been described in early stage CKD [18]. Compared with normotensive and hypertensive controls, patients with CKD stages 2–5 had significantly larger internal carotid artery diameters but comparable intima-media thickness, resulting in significantly increased circumferential wall stress [18]. Furthermore the recent Nephrotest study [19] identified aPWV velocity as an independent predictor of all cause mortality and fatal or non-fatal cardiovascular events in 439 patients with CKD stages 3–5. The same study demonstrated that the addition of PWV to traditional risk factors significantly improved the risk stratification for all-cause mortality. Arterial stiffness in early CKD patients has been associated with the presence of diastolic dysfunction [20] and increased LV mass [21]. However its relationship with systolic function remains poorly investigated and largely unknown. Evaluation of systolic function in CKD patients has traditionally been limited to volume-based assessment of LVEF and assessment of regional wall motion or visual estimation of regional thickening. Although LVEF is one of the most powerful echocardiographic predictors of death or cardiovascular morbidity is not a sensitive marker of global LV systolic function in the presence of LV hypertrophy (commonly present in CKD patients) [22]. Additionally, LVEF may be insufficiently sensitive to identify mild degrees of systolic dysfunction as proven by its inability to identify a gradation of risk in patients with EF >45 % [23]. This suggests that asymptomatic CKD patients can have myocardial dysfunction with preserved LVEF. Deformation imaging using STE may overcome this limitation. Speckle tracking echocardiography follows the motion of myocardial tissue throughout the cardiac cycle by tracking acoustic reflections, known as speckles, in previously obtained echocardiographic images. Recently two studies explored the relationship of GLS, a marker of subclinical systolic dysfunction and arterial stiffness. Krishnasamy et al. demonstrated an independent association between GLS, assessed by STE, and aPWV in patients with CKD stages 3–5 [24]. Kim and colleagues also found an independent association between brachial-ankle PWV and GLS [25]. Concurring to our findings the authors identified a close interaction between arterial stiffness and LV function. However the aforementioned studies are limited by the fact that novel elements of deformation such as LV twist were not investigated.

Alterations in LV twist have been linked with subclinical myocardial dysfunction in a variety of clinical settings [26–28], nonetheless its association with arterial stiffness has never been studied. To our knowledge the current study is the first to find an independent association between LV twist and aPWV. With increased aPWV, the reflected waves return earlier, impacting on the central arteries during systole rather than diastole thus amplifying aortic and ventricular systolic pressures. As a consequence myocardial pressure load and oxygen consumption increase, leading to subendocardial injury [2, 3]. Myocardial fiber orientation changes continuously from a right-handed helix in the subendocardium to a left-handed helix in the subepicardial region [9]. Subepicardial layers dominate the overall rotation/twist during ejection and remain preserved in early myocardial disease [9]. Subendocardial fibres counteract subepicardial dominance and are the most vulnerable and sensitive to the presence of early myocardial disease. Subendocardial injury would therefore exaggerate overall LV twist as the subepicardial fibres would function in the absence of the counteracting subendocardial ones. A similar mechanism has been identified in patients with diastolic heart failure [29]. The current findings highlight that the cardiovascular system should always be viewed as an entity. When subclinical abnormalities are detected in the heart one should also consider exploring the presence of early disease in the peripheral arterial system and vice versa. Patients with isolated hypertension [30], heart failure with normal LVEF [31], diabetes mellitus [32] or CKD [2] could potentially benefit of STE or aPWV for early identification of CVD. However, it should be noted that although STE has been validated against sonomicrometry and CMR, vendor reproducibility [33], the need for optimal image quality and time constraint are limiting STE use in routine clinical practice. However, strain imaging using STE could be used as a supplementary diagnostic method in several conditions [34]; (i) to identify early systolic dysfunction in patients with normal or preserved LVEF [8, 35], (ii) to identify subclinical LV dysfunction in individuals who are evaluated for cardiomyopathy [36], (iii) in addition to LVEF in patients undergoing chemotherapy to identify sub-clinical LV dysfunction [37].

Future randomized control studies are required to assess the potential benefit of early, aggressive risk factor management in asymptomatic CKD patients with abnormal LV twist/aPWV indices. For the time being, physicians should consider initiation of risk factor modification therapies in CKD patients with abnormal LV twist or PWV parameters, even in the absence of high CVD risk using established calculators (Q-risk, Framingham, etc).

## Limitations

Firstly due to the observational design of the study we cannot determine causality between subclinical 2DSTE abnormalities and aPWV and our results should be viewed as hypothesis generating. Secondly, even though patients recruited in the current study were asymptomatic from the cardiovascular point of view, with no previous history of ischaemic heart disease and a structurally normal heart on transthoracic echocardiogram, the presence of significant coronary artery disease was not ruled out with a functional or invasive test. However, in a subset of 20 pre-dialysis patients who subsequently underwent elective invasive angiography as part of their pre-transplant assessment, the vast majority 19 (95 %), had no significant disease in their epicardial arteries. This fact supports the hypothesis that microvascular ischaemia may be accountable for the LV twist patterns observed in our CKD population.

## Conclusions

In summary the current study provides for the first time a link between LV twist assessed by STE and early peripheral arterial disease, in asymptomatic CKD patients with preserved LVEF. Future studies of patients with these early subclinical markers of CVD should be undertaken, randomizing their participants to aggressive risk factor management versus watchful waiting. If early risk factor modification in patients with abnormal LV twist and PWV values improves outcomes, these markers could be used for risk stratification purposes in this population.

## Abbreviations

aPWV: Aortic pulse wave velocity
CKD: Chronic kidney disease
CVD: Cardiovascular disease
GLS: Global longitudinal strain
ICC: Intraclass correlation coefficients
LV: Left ventricle
LVEF: Left ventricular ejection fraction
LVMI: LV mass index
STE: Speckle-tracking echocardiography
