# Erratum to: ‘Left ventricular twist mechanics and its relation with aortic stiffness in chronic kidney disease patients without overt cardiovascular disease’

**DOI:** 10.1186/s12947-016-0059-2

**Published:** 2016-04-19

**Authors:** Samir Sulemane, Vasileios F. Panoulas, Klio Konstantinou, Athanasios Bratsas, Julia Graspa, Frederick W. Tam, Edwina A. Brown, Petros Nihoyannopoulos

**Affiliations:** 1 Imperial College London, National Heart and Lung Institute, London, UK; 2 Imperial College Healthcare NHS, Hammersmith Hospital, London, UK; 3 Imperial College, London, UK; 4 Imperial College Renal and Transplant Centre, Hammersmith Hospital, London, UK

After the publication of this work [1], it was brought to the authors’ attention that the author list on the final manuscript was incomplete.

It read as Samir Sulemane, Vasileios F. Panoulas, Klio Konstantinou, Athanasios Bratsas, Frederick W. Tam, Edwina A. Brown and Petros Nihoyannopoulos.

The correct author list should have included Dr Graspa and is now included here.

Samir Sulemane, Vasileios F. Panoulas, Klio Konstantinou, Athanasios Bratsas, Julia Graspa, Frederick W. Tam, Edwina A. Brown and Petros Nihoyannopoulos.
